# Study on the Properties and Process Parameters of Different Clays in Disc Granulation

**DOI:** 10.3390/ma13071714

**Published:** 2020-04-06

**Authors:** Hui Li, Jingjie Zhang, Wukui Zheng, Tian Cui, Yuxuan Yang

**Affiliations:** 1 College of Materials Science and Engineering, Xi’an University of Architecture and Technology, Xi’an 710055, Shaanxi, China; 15388618192@163.com (J.Z.); zheng.wukui@xauat.edu.cn (W.Z.); ctbryant@126.com (T.C.); yyx9202@163.com (Y.Y.); 2 Shaanxi Ecological Cement & Concrete Engineering Technology Research Center, Xi’an 710055, Shaanxi, China

**Keywords:** clay, disk granulation, granulated fraction, particle size, lightweight aggregate, ceramsite

## Abstract

Sintering solidification is an effective way to treat soil contaminated with nonvolatile heavy metal. The ceramsite prepared from contaminated soil after sintering can be used as lightweight aggregate in concrete. The preparation process of ceramsite can be divided into two steps: granulation and sintering. As one of the key processes, granulation is directly related to the final solidification and physical properties of ceramsite, and the properties of the clay are directly related to the granulation process. In this work, clays from different regions granulated with disc granulation were studied and compared. The results showed that different clays had significantly different performances in granulation with the same granulation system, and each clay had its own best process parameter. The significance analysis showed that the volume surface mean diameter and the reduction ratio had the most significant impact on the granulated fraction among all the factors. No matter which process parameter was used, as the particle size increased, the granulated fraction increased first and then decreased, and the best results were obtained when the average volume diameter was about 20.5 µm. Furthermore, as the reduction ratio increased, the granulated fraction decreased. These two factors are easy to measure and can be used for predicting the granulation effect of different clays, which can further guide industrial production.

## 1. Introduction

Heavy metal pollution is a serious problem in contaminated soil [1], and the polluted soil needs to be properly repaired before it can be reused. There are many possible methods of remediation, including physical remediation [2,3], chemical remediation [4,5], and biological remediation [6,7,8,9]. No matter what technology is used, the basic concept is to remove heavy metals from the soil or to reduce the biological toxicity and migration of heavy metals in the soil. Sintering solidification is one of the most effective ways to treat soil contaminated with nonvolatile heavy metal. At high temperatures, the heavy metal elements form new stable crystals or are immobilized in lattices [10,11]. Moreover, the ceramsite prepared after clay sintering can be used as lightweight aggregate in concrete [12], which can further solidify heavy metals.

The preparation of ceramsite by sintering can be divided into two steps: granulation and sintering. As one of the key processes, granulation is directly related to the final solidification and physical properties of ceramsite, and the properties of the clay are directly related to the granulation process. Clays that come from different regions and environments have huge differences in mineral composition, physical properties, and so on [13,14,15,16,17]. Thus, the granulation process of different clays may be very different. Although there has been some research about granulation, such as research on the process parameters of disc granulation [18,19,20,21,22], it differs between different granulation processes or even the mechanism of granulation [23,24,25,26]. There have been few studies on the influence of different clays on granulation.

In this work, clays from different regions in China were studied with the disc granulation system. By testing their granulation effect, the process parameter for each clay was analyzed. The comparison of different clays in disc granulation was also studied. Finally, a significance analysis was used to determine which factors heavily affect the granulation process. The details are shown below.

## 2. Experiment

### 2.1. Raw Materials and Equipment

**Figure 1:**
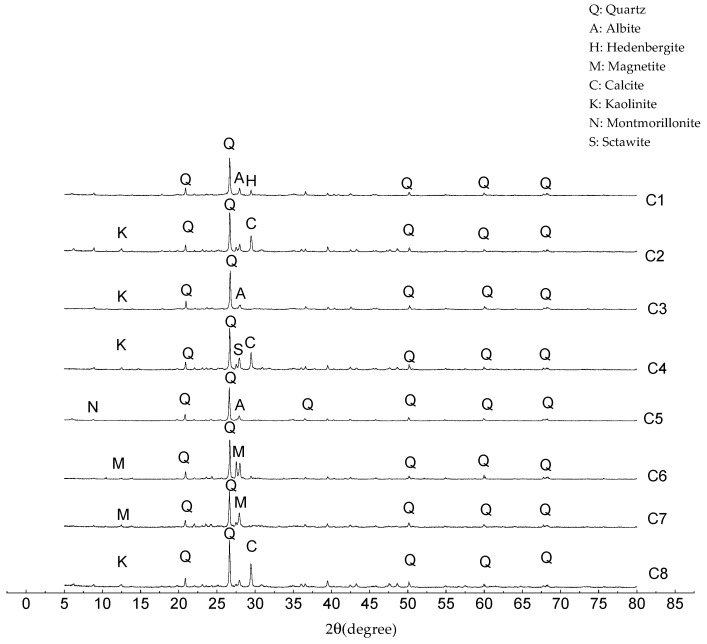
Mineralogical composition of different clays.

Eight different clays from different parts of China were collected for the experiment. Their chemical composition was analyzed by X-ray fluorescence method (XRF, Bruker, Karlsruhe, Germany), and the results are listed in Table 1. Their mineral phase composition was analyzed by X-ray diffraction (XRD, Rigaku Corporation, Tokyo, Japan), as shown in Figure 1.

Clays were collected in small blocks. Prior to use, they were put into the oven for 24 h at 105 °C (until the water content was lower than 0.5 wt.%) and then ground in a ball mill for 30 min. Finally, the samples were ready for granulation. The size distribution and volume surface mean diameter (VMD) of the different clays are shown in Table 2.

The apparent density, tap density, and reduction ratio of the different clays are shown in Table 3. Apparent density and tap density were measured by the powder comprehensive performance tester. (The mineral composition, angle of repose, and collapse angle did not significantly influence the granulation process of clay, and they are therefore not discussed in this paper.)

The equipment used in the experiment is shown in Table 4. The water used for granulation was tap water.

### 2.2. Experimental Procedures and Method

**Figure 2:**
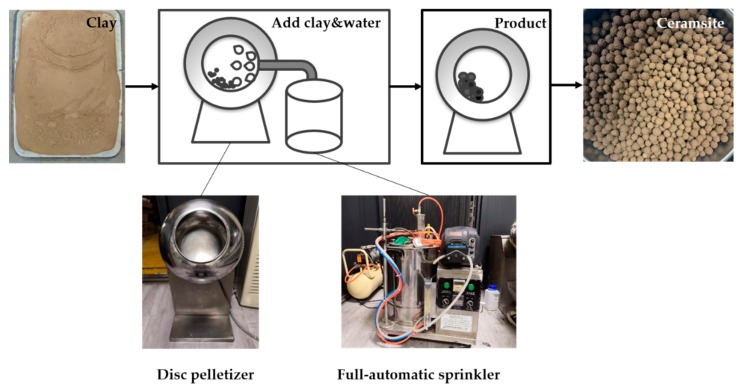
The experimental system.

In order to reduce the influence of human factors on the experiment and to reduce experimental error, an automatic spray and granulation system was used, as shown in Figure 2.

For each experiment, 1 kg of clay was used. The granulation time was set as 15 min, and the corresponding amount of water addition was controlled by setting the sprinkling time and the interval time of the sprinkler. The number of times soil materials were added equaled the number of times water was sprayed, and the amount of each addition could be calculated. 

At the beginning of the experiment, the disc granulator was turned on, and the speed was set. Clay was added into the rotating disc, the position of the sprinkler was adjusted to the same place in each experiment and aligned to the upper position of the soil center, and it was then turned on to start the granulation. After 15 min, the sprinkler was turned off, the rotational speed of the disc granulator was turned down, and the ball was then removed with a spoon at a low rotational speed. 

The main factors that influence disc granulation are the inclination angle of the disc, the amount of water added, and the rotational speed. The most suitable disc inclination is related to the diameter of the disc, that is, the disc inclination decreases with an increase in the diameter of the disc [25]. Previous experiments have shown that the granulator used in this experiment achieves the best result when the inclination angle is 55°. Therefore, the disc inclination angle was fixed to 55°. Previous experiments have also shown that the water content should be controlled in the range of 10–25%. It is difficult to make balls with clay containing too little water, while powders with too much water can easily become slurry. Based on previous experiments, the rotational speed was set between 41 and 49 r/min.

In this experiment, five kinds of clay (C1–C5) were used, and three kinds (C6–C8) were used to verify the conclusion. The influence of disc inclination, water content, and disc speed were studied according to the parameters outlined in Table 5. Each experiment with the same parameters was carried out 3 times to verify the reproducibility of the system.

### 2.3. Testing Methods

The granulated fraction was used in the analysis as it reflects the difficulty of granulation, and it was calculated by the following equation:

(1) $$\mathrm{Granulated}\;\mathrm{fraction}=\frac{\mathrm{The}\;\mathrm{amount}\;\mathrm{of}\;\mathrm{round}\;\mathrm{ball}\;\mathrm{bigger}\;\mathrm{than}\;0.9\mathrm{mm}}{\mathrm{The}\;\mathrm{amount}\;\mathrm{of}\;\mathrm{raw}\;\mathrm{material}+\mathrm{water}\;\mathrm{added}}$$

The ball size distribution was measured by screening the ball with 3, 5, 7, and 16 mesh serial sleeves. Then, the mass of different particle sizes was weighed. As this work studied the properties of different clays in the preparation of ceramsite, whether or not the particles could reach the requirement of ceramsite used as lightweight aggregate was considered the key index of granulation according to the National Standard of the People’s Republic of China “GB/T 17431.1-2010 Lightweight aggregates and its test methods-Part 1: lightweight aggregates”.

Significance analysis was carried out by SPSS® (Statistical Product and Service Solutions) software (IBM, Armonk, New York, The United States) for the properties of the different clays and the granulated fraction of each granulation process. 

## 3. Results and Discussion

**Figure 3:**
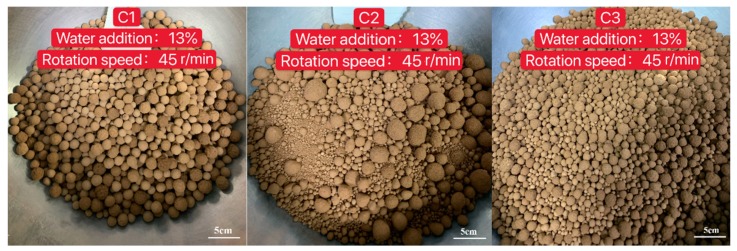
Granulation between different clays with the same process parameter.

**Figure 4:**
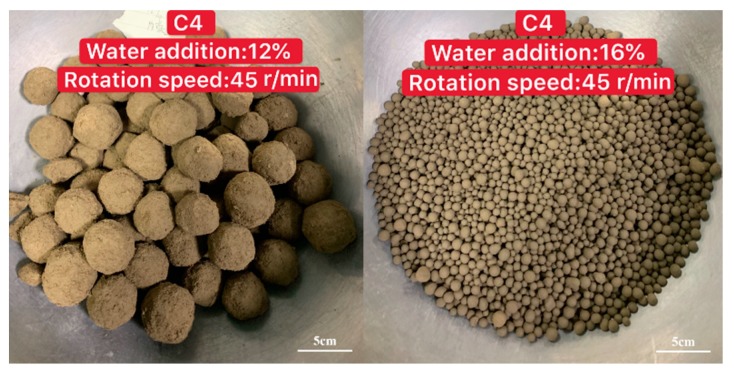
Granulation of different process parameters with the same clay.

In this experiment, it was clearly seen that all five clays from different regions could easily form balls, but the appearance and particle size distribution were significantly different. With the same process parameter, the product could form uniform balls or balls with large size distributions, as shown in Figure 3. Furthermore, with the same clay, the process had a totally different influence on the granulation results, as shown in Figure 4. Therefore, it is important to study the granulation rules of different clays and determine which factors have the greatest influence on the granulation process.

### 3.1. The Influence of Water Addition and Rotation Speed on the Granulation of Different Clays

**Figure 5:**
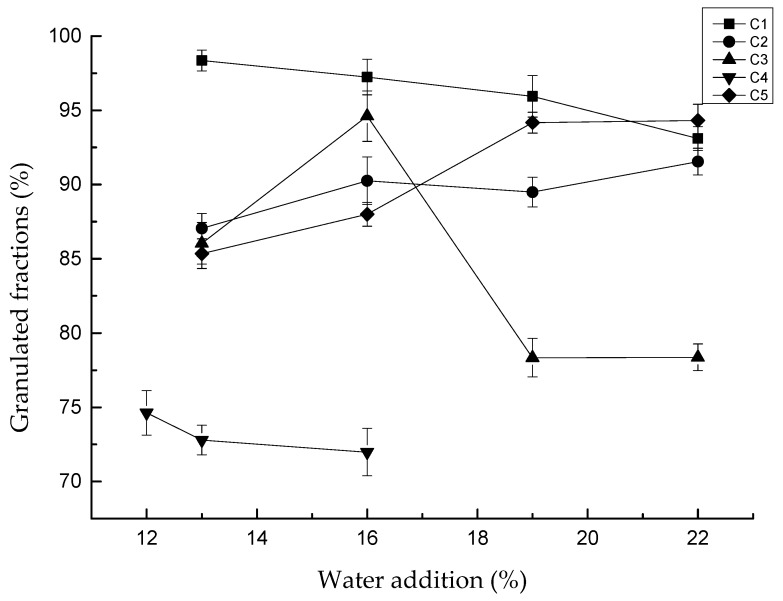
Effect of water addition on granulation (rotation speed is 45 r/min).

The influence of water addition on the granulated fraction was studied, as shown in Figure 5. For C4, when the water addition was higher than 16%, the clay turned into slurry, which could not be used for granulation. Therefore, there were only two sets of data for C4. Based on this, 12% water addition was tested for C4 in order to have more data for the analyzability of the results. 

Repeated experiments with the same parameters showed that, although the granulation size distribution was not the same every time, the difference in the fraction granulated was not big, and the tendency of forming a bigger particle was the same. This provided the basis for subsequent analysis.

The results also indicated that the effect of water addition on the granulated fraction had discrepancies with different clays. The granulated fraction of C1 decreased slightly with the increase in water content, and the highest granulated fraction was 98.35% with water addition of 13%. C2 and C5 granulated fractions increased slightly with the increase in water content, with the highest granulated fraction being 91.55% and 94.17%, respectively, with water addition of 22%. The granulated fraction of clay in C3 and C4 differed greatly with changes in water content.

**Figure 6:**
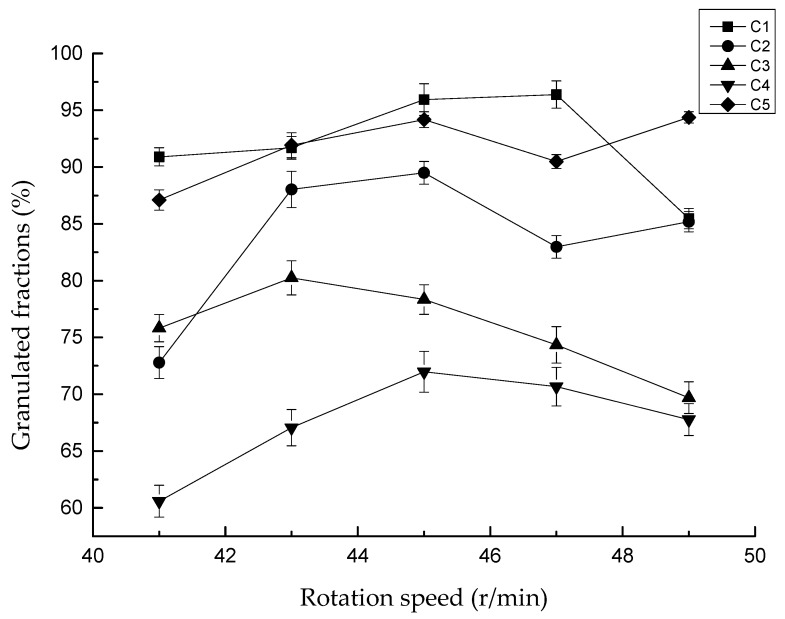
Effect of rotation speed on granulation (water addition is 13%).

The influence of rotation speed on the granulated fraction is shown in Figure 6. In general, the granulated fraction first increased and then decreased as the rotation speed increased. However, the regularity was not obvious with different clays. The granulated fraction was the highest when the disc speed was 45 r/min in C2, C4, and C5. The granulated fraction in C1 was the highest at 47 r/min, while it was the highest at 43 r/min in C3. The results also showed that the curves of the granulated fraction of different clays seldom intersected. This meant that, compared with the clay properties, the rotation speed had little influence on the granulated fraction. 

The relationship between water addition, rotation speed, and granulated fraction showed that the process parameters had an influence on the granulation results. Each clay had its own optimum granulation process, and in general, the effect of water addition on the granulated fraction was greater than rotation speed. However, the effect of the process parameters was completely different in different clays. Therefore, the process parameters for one kind of clay cannot be applied to the other. In industrial production, more suitable and generally applicable laws need to be found. 

### 3.2. Significance Analysis of Different Factors Affecting Granulation

The granulation of clay is not only influenced by the process parameter but also by its chemical composition, mineral composition, powder property, etc. In order to further understand which factors affect the granulation process of different clays, a significance analysis was carried out. 

Different variables were inputted into SPSS software to analyze the influence of changes in these factors on the granulated fraction. Besides water addition and rotation speed, the VMD, the chemical content, mineral phase content, reduction ratio, angle of repose, and collapse angle of the different clays were put into the analysis, and the significance analysis results are shown in Table 6 (the weak correlation factor has already been removed from the table).

It can be seen from Table 5 that the VMD and the reduction ratio had the most significant impact on the granulated fraction among all the factors. This means that the granulated fraction can be controlled and optimized by controlling the VMD and the reduction ratio in the granulation process.

**Figure 7:**
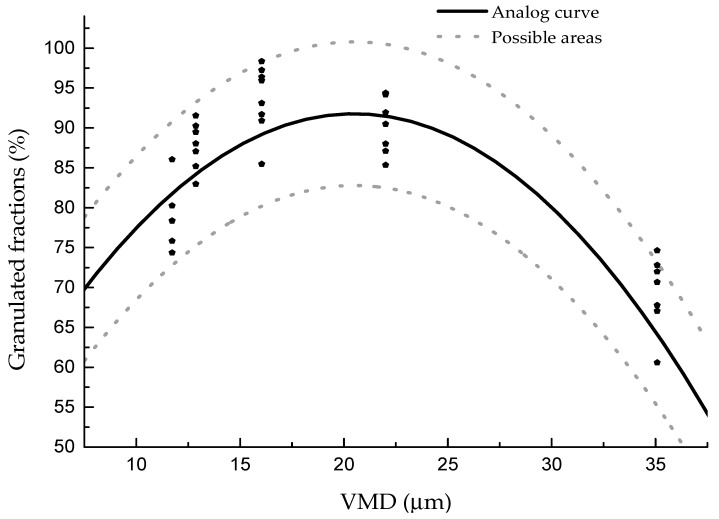
Granulated fraction of different particle sizes with different clays.

The influence of VMD on the granulated fraction with different process parameters and clays was analyzed, and the results are shown in Figure 7. It can be seen that no matter which process parameter was used, as the particle size increased, the granulated fraction increased first and then decreased. The upper and lower limits of the granulated fraction were within 10%. The formula between the granulated fraction and the VMD can be fit as follows: y = 37.15 + 5.33x − 0.13x^2^ ± 9. 

Our results were different compared to the general understanding that the smaller the particle, the easier the granulation would be. According to our formulation, clay with a VMD of 20.5 µm would be more suitable for disk granulation. This can be explained by the fact that the agglomeration of particles in disk granulation occurs mainly through the liquid bridging force, and the adhesion ability of clay particles after adding water is the main factor influencing the granulated fraction. The stronger the adhesion ability, the higher the granulated fraction. Adhesion ability is directly related to the particle size of clay. If the water added is enough to cover the surface of each particle, the larger the surface area, the easier it is to form larger particles. Therefore, with the decrease of VMD, the surface area increases, and the granulated fraction is higher. However, with further reduction of particle size to lower than 20 µm, the van der Waals force increases significantly, and particles gradually show a stronger adhesion ability [27]. Thus, particles lower than 20 µm are easily agglomerated under the action of van der Waals force to produce larger particles, resulting in decreased granulated fraction. Therefore, the granulated fraction first increased and then decreased with the increase of VMD in our experiment.

**Figure 8:**
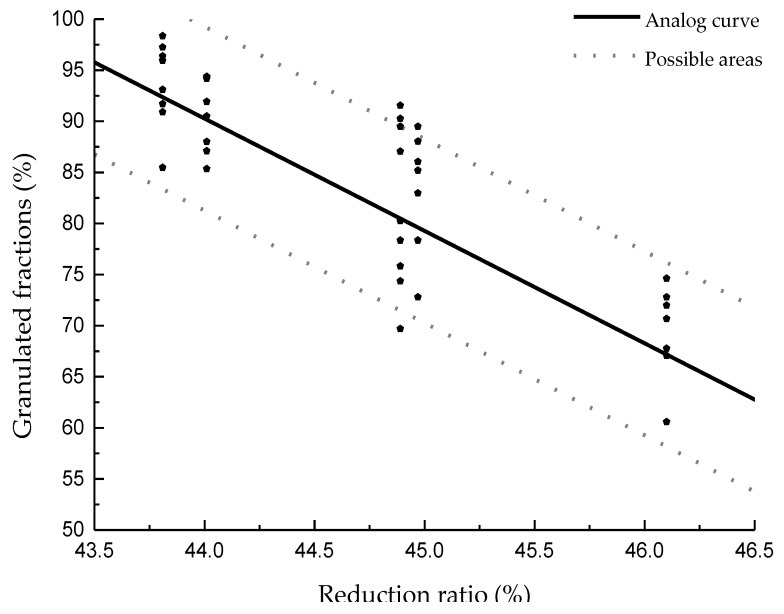
Granulated fraction of different reduction ratio compressibility with different clays.

The influence of the reduction ratio on the granulated fraction with different process parameters and clays was analyzed, and the results are shown in Figure 8. It can be seen that, as the reduction ratio increased, the granulated fraction decreased. Compared with the influence of VMD, the average granulated fraction was nearly linear. The reduction ratio reflects the flowability of the clay particles. As the flowability decreases, it becomes difficult for particles to move, resulting in difficulty in colliding and adhering to each other and causing a reduction of the granulated fraction. Thus, it is better to modify the morphology of clay particles, which will lead to a better result. The fitting formula between the granulation and reduction ratio is as follows: y = −11x + 574.27 ± 9. 

### 3.3. Validation Experiment of the Influence of VMD and Reduction Ratio on the Granulation Process

**Figure 9:**
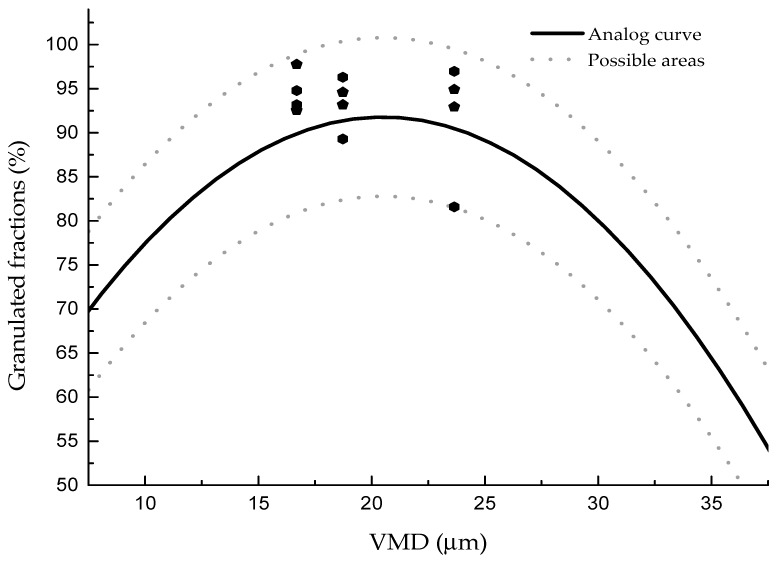
The VMD verification results. (The clays were used for granulation at a rotation speed of 45 r/min with water addition of 13%, 16%, 19%, and 22%.)

**Figure 10:**
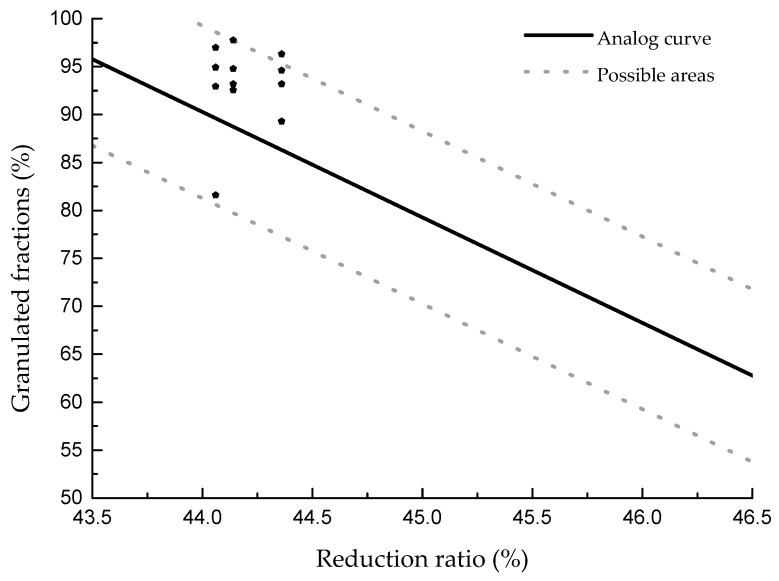
The reduction ratio verification results. (The clays were used for granulation at a rotation speed of 45 r/min with water addition of 13%, 16%, 19%, and 22%.)

In this study, in order to confirm that the curve drawn and the fitting formula are in agreement with the actual results, three groups of new clays were selected to verify the results. The new relationship between the VMD and the granulated fraction is shown in Figure 9, while the relationship between the reduction ratio and the granulated fraction is shown in Figure 10.

**Figure 11:**
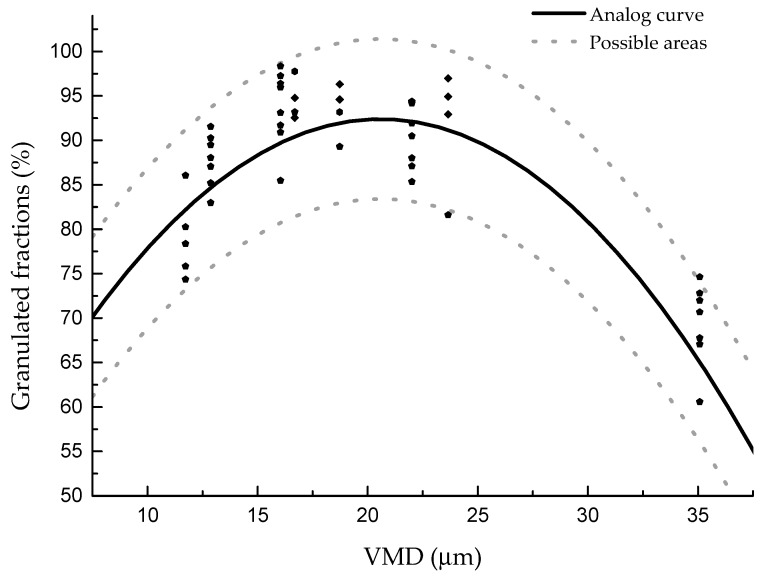
The relationship between the VMD and the granulated fraction.

**Figure 12:**
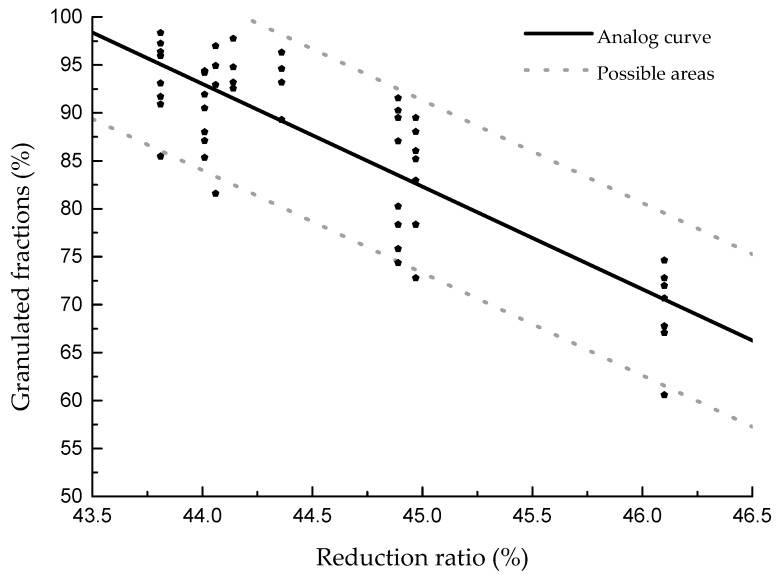
The relationship between the reduction ratio and the granulated fraction.

From Figure 9, it can be seen that the relationship between the granulated fraction and the VMD of different clays is in agreement with the previously summarized regulation, and the highest granulated fraction is slightly higher than what was previously predicted. According to the newly added data, the formula can be modified to y = 37.35 + 5.35x − 0.13x^2^ ± 9, as shown in Figure 11. In the figure, 95% of the results are within the formula area. Figure 12 shows the same results as Figure 10; the reduction ratios of the new clays are also in agreement with the previous regulation. Furthermore, by adding the new data, the formula can be modified to y = −10.7x + 563.82 ± 9, as shown in Figure 12.

The formulas of the VMD, the reduction ratio, and the granulated fraction are much more suitable and are generally applicable laws for the granulation process. They can be used for predicting the granulation effect of different clays because particle size and powder properties are relatively easy to measure. They can also be used to determine the best grinding time for raw material, which can be used to guide the actual production process. If the raw material particle size is closer to the optimal particle size from the beginning of the pretreatment stage, the production efficiency will be improved.

## 4. Conclusions

In this work, clays from different regions were used to study their differences in the disc granulation process. The influence of rotation speed and water addition on the granulated fraction were investigated. The results showed the following:

(1) The granulation performances of clay from different places were totally different. Each clay had its own optimum granulation process. The study of the clay granulation process is not representative and cannot be used to guide production.
(2) Significance analysis showed that the VMD and the reduction ratio had the most significant impact on the granulated fraction among all the factors. No matter which process parameter was used, as the particle size increased, the granulated fraction increased first and then decreased, and the best result was obtained when the average volume diameter was about 20.5 µm. Furthermore, as the reduction ratio increased, the granulated fraction decreased.

In this study, only eight different clays were used for the experiment. Thus, in the future, research should include more types of clay from different places as well as other materials; for example, industrial solid waste should be studied too. It is hoped that more rules in granulation will be found to guide the actual production process.

## Figures and Tables

**Table 1:** Chemical composition of different clays (wt.%).

Clay	Na_2_O	MgO	Al_2_O_3_	SiO_2_	K_2_O	CaO	TiO_2_	Fe_2_O_3_	Other
**C1** (Xi’an Huxian)	1.42	2.27	17.52	62.61	3.30	1.70	0.82	6.72	3.63
**C2** (Xi’an Jingyang)	0.89	3.32	12.59	46.50	2.46	22.78	0.72	5.71	5.05
**C3** (Shaanxi Hanzhong)	1.04	2.03	18.51	63.04	3.28	1.39	0.83	7.11	2.79
**C4** (Neimenggu Baotou)	2.27	2.31	13.74	60.17	2.87	8.44	0.63	5.42	4.14
**C5** (Shandong Zoucheng)	2.14	1.52	15.93	65.89	2.68	1.87	0.76	5.33	3.88
**C6** (Sichuan Luzhou)	1.93	1.32	13.09	66.48	2.43	2.05	0.91	7.99	3.81
**C7** (Heilongjiang Mohe)	1.75	1.13	18.04	60.89	2.85	1.83	1.15	8.20	4.17
**C8** (Henan Sanmenxia)	1.44	2.66	14.00	58.38	2.85	10.10	0.75	5.53	4.30

**Table 2:** The size distribution of different clays.

Clay	VMD/µm	d_10_/µm	d_50_/µm	d_90_/µm
C1	16.04	1.29	7.84	42.59
C2	12.87	1.09	5.39	35.43
C3	11.73	1.09	5.10	32.86
C4	35.08	1.78	23.90	85.81
C5	22.01	1.52	12.93	56.75
C6	23.65	1.43	11.26	67.45
C7	16.69	1.36	8.02	45.42
C8	18.73	1.34	10.58	48.25

**Table 3:** The apparent density, tap density, and reduction ratio of different clays.

Clay	Apparent Density (g/mL)	Tap Density (g/mL)	Reduction Ratio/%
C1	0.781	1.390	43.81
C2	0.750	1.363	44.89
C3	0.734	1.332	44.97
C4	0.898	1.666	46.10
C5	0.767	1.370	44.01
C6	0.829	1.482	44.06
C7	0.691	1.283	44.14
C8	0.825	1.483	44.36

**Table 4:** The equipment used in the experiment.

Equipment	Model	Company
XRF	S4 PIONEER	Brook AXS, Germany
Powder comprehensive performance tester	BT-1000	Dandong Baite Instrument Co. Ltd
Laser particle sizer	HELOS/BR/OM/RODOS/T4-R4	SYMPATEC GmbH
Drying oven	FN101-A	Changsha Instrument Factory
ball mill	Φ300×300	Shenyang North Instrument Factory
Disc pelletizer	QLC400-II	Chuzhou Huaye Electromechanical Company
Fully automatic sprinkler	GPY-01	Chuzhou Huaye Electromechanical Company

**Table 5:** The experimental method.

Experimental Method	Parameters	C1	C2	C3	C4	C5
Testing of different water addition	Inclination (degrees)	55				
	Rotation speed (r/min)	45				
	Water addition (%)	13, 16, 19, 22				
Testing of different rotation speeds	Inclination (degrees)	55				
	Water addition (%)	13				
	Rotation speed (r/min)	41, 45, 47, 49				

**Table 6:** Significance analysis results of different properties of clay.

Properties		Significance Analysis Results	
VMD	Pearson correlation		−0.488 **
	Significance		0.001
Reduction ratio	Pearson correlation		−0.825 **
	Significance		0.000

** At the 0.01 level (double tail), the correlation was significant. VMD: volume surface mean diameter.
